# Thiazolidinedione-Induced Fluid Retention: Recent Insights into the Molecular Mechanisms

**DOI:** 10.1155/2013/628628

**Published:** 2013-03-18

**Authors:** Jerzy Bełtowski, Jolanta Rachańczyk, Mirosław Włodarczyk

**Affiliations:** Department of Pathophysiology, Medical University of Lublin, 8 Jaczewskiego, 20090 Lublin, Poland

## Abstract

Peroxisome proliferator-activated receptor-**γ** (PPAR**γ**) agonists such as rosiglitazone and pioglitazone are used to improve insulin sensitivity in patients with diabetes mellitus. However, thiazolidinediones induce fluid retention, edema, and sometimes precipitate or exacerbate heart failure in a subset of patients. The mechanism through which thiazolidinediones induce fluid retention is controversial. Most studies suggest that this effect results from the increase in tubular sodium and water reabsorption in the kidney, but the role of specific nephron segments and sodium carriers involved is less clear. Some studies suggested that PPAR**γ** agonist stimulates Na^+^ reabsorption in the collecting duct by activating epithelial Na^+^ channel (ENaC), either directly or through serum and glucocorticoid-regulated kinase-1 (SGK-1). However, other studies did not confirm this mechanism and even report the suppression of ENaC. Alternative mechanisms in the collecting duct include stimulation of non-ENaC sodium channel or inhibition of chloride secretion to the tubular lumen. In addition, thiazolidinediones may augment sodium reabsorption in the proximal tubule by stimulating the expression and activity of apical Na^+^/H^+^ exchanger-3 and basolateral Na^+^-HCO_3_^−^ cotransporter as well as of Na^+^,K^+^-ATPase. These effects are mediated by PPAR**γ**-induced nongenomic transactivation of the epidermal growth factor receptor and downstream extracellular signal-regulated kinases (ERK).

## 1. Introduction

Thiazolidinediones (TZD) are synthetic exogenous agonists of peroxisome proliferator-activated receptor-*γ* (PPAR*γ*) and are used in the treatment of type 2 diabetes mellitus (T2DM). Currently, two TZDs, rosiglitazone (RGZ) and pioglitazone (PGZ), are available, although rosiglitazone is being withdrawn from the market in Europe and its use is restricted in the USA due to concerns about the increase in prevalence of myocardial infarction in RGZ-treated patients demonstrated in several clinical trials. TZDs increase insulin sensitivity, reduce blood glucose and hemoglobin A1c levels, inhibit adipose tissue lipolysis, and favorably affect adipose tissue hormones (adipokines), decrease microalbuminuria, inhibit inflammation, reduce blood pressure, and counteract hepatic steatosis and fibrosis in experimental animals and in TZD-treated patients [1–3]. However, these medications are not devoid of adverse effects among which fluid retention and edema are among the most important [4, 5]. Thiazolidinediones induce peripheral edema in 5–10% of patients if used in monotherapy and in 15–20% of those cotreated with insulin. Edema results from fluid retention manifested as the increase in body weight and total body water, small but significant 6-7% increase in plasma volume, and reduction of hematocrit, hemoglobin, and serum albumin concentrations. The prevalence and severity of edema are similar in RGZ- and PGZ-treated patients. TZD-induced edema is usually peripheral. However, these medications may precipitate or aggravate congestive heart failure which is a common comorbidity in diabetic patients, and pulmonary edema in TZD-treated patients has also been occasionally reported. TZD-induced fluid retention is often resistant to diuretics and is relieved only by drug withdrawal. The mechanisms and consequences of TZD-induced fluid retention have been described in several previous articles [6–14]. In this paper, we will focus on recent findings about the effects of TZDs on sodium handling in the kidney. These findings indicate that the mechanism of TZD-induced fluid retention is much more complex and controversial than initially appreciated.

## 2. Renal Sodium Handling: An Overview

Although vascular effects, that is, vasodilation and increase in transendothelial permeability, may contribute to thiazolidinedione-induced edema and fluid retention, there is little doubt that alterations of renal Na^+^ handling by the kidney play a key role. In the kidney, Na^+^is first filtered in glomeruli and then >99% of it is reabsorbed in renal tubules. Sodium reabsorption occurs throughout the nephron with 60–70% of filtered Na^+^ being reabsorbed in the proximal tubule (PT), 20–25% in the medullary thick ascending limb of Henle's loop (mTAL), 5–10% in distal convoluted tubule (DCT), and the rest in the collecting duct (CD). Sodium reabsorption consists of two steps. First, Na^+^enters the tubular cell through the apical (luminal) membrane carriers which vary along the nephron. In the proximal tubule Na^+^ reabsorption is accounted for by Na^+^/H^+^ exchanger-3 (NHE3), Na^+^-phosphate cotransporter-2 (Na-Pi2), Na^+^-glucose, and Na^+^-amino acid cotransporters. In the mTAL, DCT, and CD apical sodium reabsorption is driven by loop diuretics-sensitive Na^+^-K^+^-2Cl^−^ cotransporter (NKCC), thiazide-sensitive Na^+^-Cl^−^ cotransporter (NCC), and amiloride-sensitive epithelial sodium channel (ENaC), respectively, although these carriers overlap to some extent at transition from one segment to the other. In contrast, the second, active step of Na^+^ transport, that is, its extrusion from tubular cell to the peritubular space, is always driven by sodium-potassium adenosine triphosphatase (Na^+^,K^+^-ATPase) irrespectively of the nephron segment. 

Although even very minor changes in glomerular filtration rate (GFR) may have profound effects on overall Na^+^ excretion, it is generally assumed that regulation of sodium balance is mainly regulated at the level of tubular reabsorption. The latter process is regulated by a myriad of neurohormonal factors which either stimulate reabsorption and decrease natriuresis, such as norepinephrine, angiotensin II, aldosterone, glucocorticoids, and insulin, or have the opposite effects such as nitric oxide (NO), angiotensin (1-7), prostaglandins, bradykinin, dopamine, and cardiac natriuretic peptides. Any compound administered systemically may affect renal Na^+^ handling either directly, by modulating tone of afferent and efferent vessels (and thus affecting GFR) or tubular function, or, indirectly, by modulating these neurohormonal systems. In contrast, if effect of a given compound on isolated tubule segments or tubular cells is examined, only direct mechanisms will be operative. Therefore, we will review separately *in vivo* and *in vitro* studies aimed to elucidate renal effects of thiazolidinediones.

## 3. Effect of TZDs on Renal Sodium Handling: *In Vivo* Studies

If anything general about effect of TZDs on renal Na^+^ handling can be said, it will be that this effect is controversial with regard to both tubular segment and specific carriers involved. A potent PPAR*γ* agonist, farglitazar (GI262570), administered at 8 mg/kg for 10 days induced plasma volume expansion in normal healthy rats as evidenced by significant reduction of hematocrit, hemoglobin, and serum albumin concentration but had no effect on GFR, renal blood flow, or filtration fraction. These results suggest that GI262570 induced fluid retention by tubular rather than glomerular mechanism [15]. In the subsequent study [16], GI262570 administered at 2 or 20 mg/kg/day for 5 days increased plasma Na^+^ and Cl^−^ and reduced K^+^ concentration, which suggests its effect on Na^+^ transport in the distal nephron where Na^+^ reabsorption is coupled to K^+^ excretion. In addition, upregulation of the expression of several genes associated with tubular transport, such as *α*-1 subunit of Na^+^,K^+^-ATPase, serum- and glucocorticoid-stimulated kinase-1 (SGK-1), glucocorticoid receptor, and aquaporin 2 (AQP2), was observed in the renal medulla of GI262570-treated rats. This PPAR*γ* agonist also tended to increase, although not significantly, the expression of *α*-subunit of ENaC but had no effect on its *β* and *γ* subunits as well as on the mineralocorticoid receptor, AQP3, and vasopressin receptor-2 (AVP-R2). Moreover, GI262570 had no effect on the expression of 11*β*-hydroxysteroid dehydrogenase, the enzyme which converts active cortisol to the inactive cortisone in the kidney and protects mineralocorticoid receptors from being activated by cortisol. The expression of natriuretic peptide receptor A (NPR-A) tended to be lower in GI262570-treated rats. Finally, PPAR*γ* agonist reduced plasma aldosterone concentration by about 40%, which is most likely secondary to extracellular fluid expansion in these animals [16]. 

Song et al. [17] have demonstrated that administration of rosiglitazone to male Sprague-Dawley rats at 94 mg/kg for 3 days decreased urine output and urinary sodium excretion by 22% and 44%, respectively. Creatinine clearance, a marker of glomerular filtration rate, was lower in RGZ-treated than in control animals by 35%, whereas fractional sodium excretion (the ratio between Na^+^ excretion and the amount of Na^+^ filtered) did not change. These data suggest that RGZ decreased sodium excretion primarily by reducing GFR while having little effect on its tubular transport. However, rosiglitazone increased whole-kidney expression of several sodium carriers including *α*-1 subunit of Na^+^,K^+^-ATPase, NHE3, Na-Pi2, and NKCC by 82%, 50%, 30%, and 97%, respectively. In contrast, the expression of other carriers including NCC, *α*-, *β*-, and *γ*-subunits of ENaC did not change. Given the distribution of these carriers along the nephron, these data indicate that if RGZ has any effect on tubular transport, this effect is exerted in the proximal tubule or the loop of Henle rather than in the distal tubule or the collecting duct. Moreover, rosiglitazone increased the expression of AQP2 and AQP3 but had no effect on AQP1 in whole-kidney homogenates. Because the expression of AQP4 in the whole kidney was below detection limit, the expression of some carriers was also assessed separately in the inner medulla. Herein, RGZ increased protein level of AQPs1-3 but not of AQP4 and had no effect on *α*-1 subunit of Na^+^,K^+^-ATPase [17]. Authors concluded that rosiglitazone, at least when administered for a short time, induces positive Na^+^ balance by decreasing GFR and possibly stimulating Na^+^ reabsorption in the proximal tubule [17]. Stimulation of Na^+^ reabsorption may be a compensatory response to the decrease in blood pressure which was observed in RGZ-treated rats driven by sympathetic and/or renin-angiotensin-aldosterone system, although the latter mechanism was unlikely because plasma renin activity as well as aldosterone concentration did not differ between control and RGZ-treated groups. Interestingly, although rosiglitazone had no effect on urinary excretion of NO metabolites, nitrites + nitrates (NO_*x*_), the ratio between urinary NO_*x*_ and creatinine concentration tended to increase and the expression of endothelial NO synthase (eNOS) in the kidney was by 33% higher in RGZ-treated rats. Because in the kidney NO increases GFR and inhibits tubular Na^+^ reabsorption, these results indicate that downregulation of NO is not involved in antinatriuretic effect of rosiglitazone [17].

The different conclusions from above-mentioned studies, that is, TZDs induce fluid retention by primarily tubular [15, 16] or glomerular [17] mechanisms, may result from using different PPAR*γ* agonists (farglitazar versus rosiglitazone) as well as time and dose of agonist administration. In particular, short-term administration of rosiglitazone reduced GFR [17]; the effect which might disappear after prolonged treatment due to increase in extracellular fluid volume as well as systemic adaptive neurohormonal mechanisms.

In healthy human volunteers, pioglitazone (45 mg/day for 6 weeks) had no effect on insulin sensitivity, blood pressure, GFR, or renal blood flow on either low- or high-salt diet. However, pioglitazone reduced sodium excretion and increased fractional Na^+^ reabsorption in the proximal tubule (measured on the basis of lithium clearance) on both low- and high-salt diet [18]. These results support primary tubular effect of thiazolidinediones.

It should be noted that antinatriuretic effect of TZDs is specific for insulin sensitive lean animals. Under physiological conditions, locally produced dopamine decreases Na^+^,K^+^-ATPase activity in the proximal tubule through the mechanism involving D1 receptors and G_s_ protein/adenylyl cyclase/cAMP and G_q_ protein/phospholipase C pathways. This effect is impaired in various experimental models of insulin resistance and hyperinsulinemia such as leptin receptor defective obese Zucker rats or rats with obesity induced by high-calorie diet in which reduced density of D1 receptors and/or their coupling to downstream G proteins is observed. In these animals, TZDs, by increasing insulin sensitivity and reducing serum insulin level, restore dopamine-induced inhibition of sodium pump, improve Na^+^ excretion, and decrease blood pressure [19–21]. Thus, thiazolidinediones may restore natriuretic effect of dopamine in insulin-resistance states.

## 4. The “ENaC Hypothesis”: Studies in Mice Lacking PPAR*γ* in the Collecting Duct

The study of Guan et al. published in 2005 suggested that TZDs increase Na^+^ reabsorption in the collecting duct by activating epithelial sodium channel. In mice lacking PPAR*γ* locally in the collecting duct neither rosi- nor pioglitazone induced a significant increase in body weight or total body water content, and ENaC inhibitor, amiloride, blocked PGZ-induced fluid retention in wild-type animals [22]. In addition, untreated mice lacking PPAR*γ* in the collecting duct excreted more sodium in their urine despite having twofold higher aldosterone level than wild-type mice, suggesting that endogenous PPAR*γ* agonists may regulate sodium balance by acting locally in the collecting duct. In CD cells isolated from wild-type mice, pioglitazone stimulated amiloride-sensitive apical-to-basolateral Na^+^ flux and the expression of *γ*ENaC mRNA. Interestingly, whereas during first 18 hours after adding PGZ, only amiloride-sensitive but not amiloride-insensitive Na^+^ flux increased suggesting specific effect of TZD on ENaC-driven Na^+^ reabsorption; after that time also amiloride-insensitive Na^+^ flux was higher in PGZ-treated cells indicating that additional mechanisms appear [22].

Interestingly, *γ*ENaC was most likely the direct PPAR*γ* target gene in this study [22]. Indeed, 4 putative PPREs were identified in the intron 1 of the *γ*ENaC-encoding gene, and in genomic DNA isolated from mice CD cells; PPAR*γ* was able to physically interact with this gene. In addition, increase in *γ*ENaC-encoding mRNA after PGZ treatment was insensitive to translation inhibitor, puromycin, indicating that synthesis of *γ*ENaC is not secondary to the regulation of any other protein. Thus, the most likely possibility is that PPAR*γ* directly binds to the PPRE and stimulates *γ*ENaC transcription. In contrast to *γ*ENaC which was 10-fold higher after PGZ treatment, no changes in *α*- or *β*-subunits of the channel were observed. Interestingly, basal (in the absence of pioglitazone) amiloride-sensitive Na^+^ flux was higher in CD cells lacking PPAR*γ*, suggesting that unliganded receptor decreases the expression of its target gene; the phenomenon is not uncommon for other nuclear receptors as well [22].

Similarly, CD-specific knockout of PPAR*γ* abolished the effect of rosiglitazone on hematocrit, plasma volume, and natriuresis *in vivo* as well as markedly attenuated its effect on Na^+^ flux in isolated CD cells *in vitro* in the other study [23]. Consequently, rosiglitazone decreased plasma aldosterone in wild-type but not in PPAR*γ* knockout mice. Taken together, the results of these two studies strongly suggested that PPAR*γ* agonists specifically stimulate Na^+^ reabsorption in the collecting duct. This conclusion is also consistent with the earlier study in human HCCD cells derived from the collecting duct [24] in which RGZ or PGZ increased cell surface expression of *α*ENaC already after 4 hours of treatment before any effect on its total protein or mRNA level was observed. The distribution of ENaC between intracellular and plasma membrane pools is regulated by SGK1—the serine/threonine protein kinase activated in the collecting duct by various factors such as aldosterone, cortisol, and insulin. ENaC contained in the plasma membrane is ubiquitinylated by E3 ubiquitin ligase Nedd2-4, endocytosed, and targeted for proteasomal degradation. SGK1 phosphorylates and inactivates Nedd2-4, thus increasing ENaC density in the plasma membrane [25]. Either PGZ or RGZ applied for 4 hours increased SGK1 activity in HCCD cells [24]. Because PPAR*γ* activates phosphoinositide 3-kinase (PI3K) in many tissues, and its downstream targets, phosphoinositide-dependent protein kinases-1 and -2 regulate SGK1 in CD cells in response to insulin, it was hypothesized that TZDs may activate SGK1 by the same mechanism. However, the activity of protein kinase B/Akt, which is a common downstream target of PI3K, was not altered by TZDs in HCCD cells excluding this possibility [24]. On the other hand, both RGZ and PGZ increased SGK1 mRNA and protein levels and this effect was abolished by PPAR*γ* antagonist, GW9662, suggesting PPAR*γ*-induced upregulation of this kinase at the transcriptional level. Bioinformatic analysis predicted six potential PPREs within the promoter region of human *sgk1* gene, and one of these PPREs (corresponding to 1801–1778 bp upstream of the SGK1 translation start) physically interacted with PPAR*γ*-RXR dimer in the electrophoretic mobility shift assay [24]. These data strongly suggest that SGK1 is a target for PPAR*γ* in the collecting duct cells. In contrast to this study, in mice CD cells PGZ did not increase the expression of SGK1 [22, 23]. Thus, although the final executor of TZD-induced Na^+^ transport is ENaC in both mice knockout and human HCCD cells studies [22–24], the precise mechanisms differ, that is, direct SGK1-independent PPAR*γ*-induced *γ*ENaC expression [22] versus SGK1-mediated redistribution of the channel to the plasma membrane [24]. Interestingly, several polymorphisms of the *β*ENaC encoding *Scnn1b* gene were associated with farglitazar-induced edema in T2DM patients suggesting the role of ENaC in fluid retention in humans as well [26].

Artunc et al. [27] compared the effect of pioglitazone (25 mg/kg/day) in wild-type (*sgk1+/+*) and* sgk1−/−* mice. Pioglitazone increased renal SGK1 mRNA and protein in* sgk1+/+* mice. The effects of PGZ on body weight and hematocrit were substantially lower and effect on plasma volume measured by the Evans blue technique was absent in s*gk1−/−* mice. PPAR*γ* expression in the kidney and plasma PGZ concentration did not differ between genotypes. Before PGZ treatment, *sgk1−/−* mice exhibited higher plasma aldosterone concentration than their wild-type counterparts. Pioglitazone treatment decreased plasma aldosterone to the similar extent in both genotypes such that after-treatment aldosterone level was still higher in the knockout mice. These data support the role of SGK1 in TZD-induced volume expansion.

## 5. ENaC-Independent Mechanisms in and Outside the Collecting Duct

Although the “ENaC hypothesis” was widely accepted several years ago, many subsequent studies indicate that it is not the only mechanism of TZD-induced fluid retention. For example, in several cultured cell lines including A6 (derived from *Xenopus laevis*), murine M-1, and mouse principal kidney cortical collecting duct (mpkCCD_c14_) neither basal nor insulin-stimulated amiloride-sensitive Na^+^ transport measured as short circuit current was affected by pioglitazone or the more potent nonthiazolidinedione PPAR*γ* agonist, GW7845, although all these cells expressed PPAR*γ* and responded to their agonist by increasing synthesis of its target, CD36. In addition, either pioglitazone or GW7845 had no effect on the expression of SGK1 in this study [28]. In contrast, long-term treatment with GW7845 significantly decreased ion transport in the M-1 cells.

In a recent study [29], a 7-day treatment with TZD induced fluid retention in mice; however, this was unexpectedly associated with a decline in *α*ENaC and *β*ENaC mRNA in the renal cortex as well as the decrease in cortical *γ*ENaC protein. Similarly, PPAR*γ* agonists decreased *α*ENaC and *γ*ENaC in murine CCD cell line M-1. Surprisingly, pioglitazone decreased *γ*ENaC promoter activity in this study. This effect did not result from direct binding of PPAR*γ* to the PPRE in the *γ*ENaC promoter region but was secondary to synthesis of other protein because it was inhibited by translation inhibitor, cycloheximide [29]. Interestingly, suppression of *γ*ENaC resulted from the decrease in histone H4K5 acetylation at the proximal promoter. Histone acetylation in general results in less compact chromatin structure and enhanced transcription. These data indicate that pioglitazone modifies chromatin structure and suppresses *γ*ENaC gene expression in this manner [29].

Vallon et al. [30] have demonstrated that collecting duct-specific inactivation of *α*ENaC had no effect on rosiglitazone- or pioglitazone-induced increase in body weight and plasma volume expansion in mice. In addition, in principal cells of the cortical collecting duct isolated from TZD-treated wild-type mice, no changes in ENaC density or open probability were observed in comparison to untreated animals, indicating that ENaC has no role in TZD-stimulated Na^+^ reabsorption. However, in primary cultures of mouse inner medullary collecting duct cells in which ENaC is virtually absent and the other type of Na^+^ channel, nonselective cyclic nucleotide-gated cation channel (NSC) predominates, pioglitazone (1 *μ*M) increased channel density by almost twofold. Authors suggested that NSC rather than ENaC is crucial for TZD-induced fluid retention.

In mpkCCD cell monolayers, rosiglitazone, pioglitazone, and troglitazone had no effect on transepithelial Na^+^ flux or ENaC density; however, two structurally distinct PPAR*γ* antagonists, T0070907 and GW9662, reduced basal as well as insulin-stimulated Na^+^ reabsorption. It is possible that high concentration of endogenous PPAR*γ* agonists maintains high intensity of Vaseline Na^+^ reabsorption and preclude TZDs from further activating Na^+^ flux in this model [31].

In Madin Darby canine kidney C7 cells (MDCK-C7) which are the experimental model of principal cells of the collecting duct, pioglitazone and GI262570 inhibited vasopressin-stimulated chloride secretion through the apical membrane by reducing the expression of cystic fibrosis transmembrane conductance regulator (CFTR) chloride channel [32]. This effect did not result from the attenuation of vasopressin-induced increase in intracellular cAMP concentration or from the inhibition of protein kinase A activation. Chloride secretion to the tubular fluid may inhibit sodium reabsorption by creating a negative intratubular potential; thus, inhibition of chloride secretion will result in the enhancement of net Na^+^ reabsorption even if Na^+^ carriers are not directly targeted [33].

Apart from the collecting duct, PPAR*γ* agonists may regulate transport in other nephron segments. In primary cultures of human proximal tubular cells, pioglitazone as well as structurally unrelated PPAR*γ* agonist, L-805646, increased NHE3, AQP1, and AQP7 expression at both mRNA and protein levels [34]. These effects were abolished by SGK1 inhibitor, GSK650394A, as well as by the silencing of this kinase by small interfering RNA.

Some clinical studies suggest that fluid-retaining effect of TZDs is more frequent in postmenopausal women. Yoshioka et al. [35] demonstrated that pioglitazone (160 mg/kg/day for 8 weeks) reduced urinary sodium excretion (−55%) only in ovariectomized but not in ovary-intact female Zucker obese rats. In male rats PGZ rather increased natriuresis in that study. Interestingly, PGZ had no effect on urinary NO_x_ excretion in either male or ovary-intact female animals but increased it in ovariectomized female rats. Pioglitazone had no effect on renal expression of eNOS and nNOS as well as on either cyclooxygenases-1 or -2 and urinary prostaglandin E_2_. In addition, pioglitazone did not modify the expression of *α*-, *β*-, or *γ*ENaC. Ovariectomy increased the expression of cytochrome P4504A isoform (CYP4A) which metabolizes arachidonic acid to 20-hydroxyeicosatetraenoic acid (20-HETE)—the derivative which induces vasoconstriction and suppresses tubular Na^+^ reabsorption. Pioglitazone had no effect on CYP4A in either male or ovary-intact female animals but reduced its expression in ovariectomized females by >64%. Thus, pioglitazone-induced sodium retention in ovariectomized females could be accounted for, at least in part, by the decrease in 20-HETE. Interestingly, PPAR*α* agonist, fenofibrate, which upregulates CYP4A in the kidney had no effect on Na^+^ excretion in rats not receiving pioglitazone, but restored CYP4A expression and completely reversed decrease in natriuresis in ovariectomized rats treated with pioglitazone [35].

## 6. PPAR*γ*-c-Src-EGFR-ERK Pathway

Recently, Endo et al. [36] published a very interesting paper in which they characterize completely new mechanism for PPAR*γ*-dependent regulation of tubular transport. In isolated rabbit, rat, and human proximal tubules, pioglitazone at very low concentration (0.3 *μ*M) stimulated basolateral Na^+^-^−^bicarbonate cotransporter (NBC) and apical NHE3 within 5 minutes. Very short time of response suggests its nongenomic nature whereas concentration range indicates that the effect may be relevant in PGZ-treated patients. These effects of pioglitazone were suppressed by PPAR*γ* antagonist, GW9662, nonspecific protein tyrosine kinase inhibitor, genistein, specific inhibitor of cytosolic tyrosine kinase c-Src, PP2, specific inhibitor of epidermal growth factor receptor (EGFR), AG1478, and extracellular signal-regulated kinase (ERK) inhibitor, PD98059, but not by either protein kinase C or protein kinase A inhibitors, calphostin C, or H89, respectively. In addition, pioglitazone increased phosphorylation level of ERK in proximal tubule cells, which was also prevented by GW9662, PP2, AG1478, and PD98059. The diffusion of cytosolic expression of PPAR*γ* was observed in rabbit, rat, and human proximal tubular cells and pioglitazone increased c-Src phosphorylation in the renal cortex of rats and rabbits, which was prevented by PP2 but not by AG1478, indicating that EGFR is downstream of c-Src in the signaling cascade [36].

EGFR is a plasma membrane tyrosine kinase which may be activated by its cognate ligand, epidermal growth factor (EGF), and several other proteins like heparin-binding epidermal growth factor (HB-EGF) [37, 38]. EGFR is highly expressed in renal tubules and its peptide ligands are abundant in urine. However, apart from its ligands, EGFR may be activated by many other factors such as angiotensin II, endothelin-1, catecholamines, and aldosterone; the phenomenon is referred to as “transactivation.” Several mechanisms of EGFR transactivation have been described among which two are the most important: (i) stimulation of metalloprotease-dependent cleavage of HB-EGF from its inactive membrane-bound precursor and (ii) direct phosphorylation of intracellular EGFR domain by c-Src. Transactivation of EGFR by these factors has been observed in blood vessels and the kidney and may contribute to the development of arterial hypertension by increasing vascular tone and/or tubular Na^+^ reabsorption [38]. Indeed, EGFR inhibitors such as AG1478 reduce vascular tone and blood pressure in several experimental models of hypertension [38, 39]. Upon activation, EGFR triggers several intracellular signaling mechanisms including (i) Ras protein, Raf-1 kinase, MEK kinase, and ERK; (ii) phosphoinositide 3-kinase; and (iii) phospholipase C*γ*. The results of Endo et al. strongly suggest that TZDs, in a PPAR*γ*-dependent manner, transactivate EGFR in the proximal tubule by activating c-Src, ultimately leading to NHE3 and NBC-driven sodium reabsorption. Indeed, *in vivo* single dose of pioglitazone (10 mg/kg), producing its plasma concentration of about 0.3 *μ*M, reduced fractional lithium clearance, urine output, and free water clearance in the rat [36]. This effect was abolished by the inhibitor of proximal tubule carbonic anhydrase, acetazolamide. 

This study [36] raises the question of how PPAR*γ* can stimulate c-Src-EGFR-ERK pathway without affecting gene expression. To answer this question, authors examined the effect of PGZ on embryonic fibroblasts from wild-type and PPAR*γ−*/*−* mice. In PPAR*γ*+/+ but not PPAR*γ−*/*−* cells, pioglitazone rapidly stimulated NHE1 and increased ERK phosphorylation, the effects which were abolished by GW9662, AG1478, PD98059, or PP2 but not by transcription inhibitor, actinomycin D, supporting its nongenomic nature [36]. Interestingly, higher PGZ concentration (30 *μ*M) decreased NHE1 activity to the similar extent in PPAR*γ*+/+ and PPAR*γ−*/*−* cells, in PPAR*γ*-independent manner. Adenovirus-mediated transfer of mice PPAR*γ* construct to cells lacking this receptor restored the ability of 0.3 *μ*M PGZ to stimulate NHE1 and to increase ERK phosphorylation. The same was observed after transfection of PPAR*γ* ligand-binding domain which is able to bind TZDs but cannot bind to DNA or stimulate gene expression. Ligand-binding domain mutant, Q284P, which cannot bind TZDs did not restore the effect of pioglitazone. These results indicate that the effect of pioglitazone requires its binding to PPAR*γ* but does not require transcriptional activity of the receptor. Pioglitazone failed to increase NHE1 activity or ERK phosphorylation in fibroblasts isolated from c-Src*−*/*−* mice, and c-Src and PPAR*γ* were coimmunoprecipitated by antibody specific for each of these proteins suggesting their direct interaction. Finally, pioglitazone increased the association of PPAR*γ* with c-Src. In contrast to wild type, ligand-binding domain mutant Q284P could not interact with c-Src [36]. Taken together, these results indicate that TZDs transactivate EGFR by inducing PPAR*γ*-c-Src interaction.

Interestingly, PGZ did not increase NBC or NHE3 activity in mice proximal tubule *in vitro* and had no effect on natriuresis and lithium clearance in this species *in vivo*. In addition, pioglitazone had no effect on either c-Src or ERK phosphorylation, most likely due to high baseline phosphorylation of c-Src in the mouse proximal tubule. These results may explain why the collecting duct appeared as the main TZD target in previous mice studies [36].

## 7. Effect of PPAR*γ* Agonists on Renal Na^+^,K^+^-ATPase

Previously, it has been demonstrated that activated EGFR can stimulate renal sodium pump in ERK-dependent manner. For example, adipose tissue hormone, leptin, increased renal Na^+^,K^+^-ATPase activity by inducing c-Src-dependent activation of the EGFR-ERK pathway [39, 40]. Because TZDs have been demonstrated to stimulate ERK and to transactivate the EGFR in nonrenal cells in some earlier studies [41, 42], before the study of Endo et al. [36] has been published, we started to examine if TZDs can regulate renal Na^+^,K^+^-ATPase activity. In our study either RGZ or PGZ was infused into the renal artery in anesthetized rats to avoid their systemic effects and to maintain *in situ* renal function. We demonstrated that both TZDs rapidly (within 15 min) increased pump activity in the renal cortex and their effect was prevented by GW9662, PP2, AG1478, and PD98059 (Bełtowski J. et al., manuscript in preparation). In addition, thiazolidinediones increased c-Src phosphorylation, EGFR phosphorylation at Tyr^845^ (the c-Src target site), and ERK phosphorylation. The effect on c-Src phosphorylation was abolished only by GW9662 and PP2, effect on EGFR phosphorylation also by AG1478, and effect on ERK phosphorylation by GW9662, PP2, AG1478, and PD98059, consistently with the existence of PPAR*γ*-c-Src-EGFR-ERK signaling cascade. In contrast, effects of TZDs were not prevented by matrix metalloprotease inhibitors or anti-HB-EGF antibodies, indicating that cleavage of HB-EGF was not involved. Because the majority of renal cortical Na^+^,K^+^-ATPase is contained in the proximal tubule, our results support the findings of Endo et al. and suggest that TZD-induced increase in Na^+^ reabsorption may be mediated not only by NBC but also by Na^+^,K^+^-ATPase; this finding is of importance since NBC drives only a minor portion of basolateral Na^+^ efflux.

## 8. Effect of PPAR*γ* Agonists in Heart Failure

Most of the experimental studies cited above were performed on healthy animals. Goltsman et al. [43] recently examined the effect of rosiglitazone administered at a dose of 30 mg/kg/day for 4 weeks in rats with congestive heart failure (CHF) induced by aortocaval fistula. Surprisingly, rosiglitazone improved sodium excretion which was impaired in CHF rats. Although SGK1 expression in the renal cortex tended to be higher in RGZ-treated animals, *α*ENaC in the renal cortex and outer medulla as well as NCC in the renal cortex was lower in RGZ-treated rats. In addition, rosiglitazone reduced the expression of angiotensin-converting enzyme in the heart and plasma aldosterone concentration. These results indicate that TZD may be more beneficial in patients with heart failure and extracellular fluid volume expansion than in those with normal volume status.

## 9. Conclusions and Future Perspectives

The mechanism of thiazolidinedione-induced fluid retention is controversial, but several consistent findings can be pointed out. First, in most studies these agents had no effect on renal hemodynamics and glomerular filtration rate suggesting principal tubular effect. Second, thiazolidinediones are unlikely to modulate renal sodium handling by affecting the regulatory neurohormonal pathways. Indeed, increase in natriuretic NO and decrease in sodium-retaining aldosterone were quite consistently reported. If any, CYP450-dependent arachidonate derivatives such as 20-HETE may be involved under some circumstances. Direct or SGK1-mediated effect on collecting duct ENaC is controversial. Some additional mechanisms such as stimulation of nonspecific cation channel in the inner medullary collecting duct, inhibition of Cl^−^ secretion, and stimulation of NHE3, NBC, and Na^+^,K^+^-ATPase in the proximal tubule may also be important. The nongenomic PPAR*γ*-c-Src-EGFR-ERK mechanism recently identified in the proximal tubule mediates rapid effect of PPAR*γ* agonists on Na^+^ transport in this nephron segment.

Although clinical use of thiazolidinediones is now limited, PPAR*γ* remains an attractive target for antidiabetic therapy. Several nonthiazolidinedione selective PPAR*γ* modulators with preserved insulin-sensitizing but reduced or absent side effects have been synthesized and are currently tested in preclinical and early-phase clinical studies. These compounds such as balaglitazone [44] are very likely to be used in the treatment of diabetes in the near future. Therefore, better understanding of renal effects of PPAR*γ* agonists is essential to optimize PPAR*γ*-based therapies.
